# Elements of the Endomucin Extracellular Domain Essential for VEGF-Induced VEGFR2 Activity

**DOI:** 10.3390/cells9061413

**Published:** 2020-06-05

**Authors:** Zhengping Hu, Issahy Cano, Kahira L. Saez-Torres, Michelle E. LeBlanc, Magali Saint-Geniez, Yin-Shan Ng, Pablo Argüeso, Patricia A. D’Amore

**Affiliations:** 1 Schepens Eye Research Institute of Massachusetts Eye and Ear, Boston, MA 02114, USA; zhengping_hu@meei.harvard.edu (Z.H.); Issahy_Cano@MEEI.HARVARD.EDU (I.C.); Kahira_Saez-Torres@MEEI.HARVARD.EDU (K.L.S.-T.); meleblan@gmail.com (M.E.L.); Magali_Saintgeniez@MEEI.HARVARD.EDU (M.S.-G.); Eric_Ng@MEEI.HARVARD.EDU (Y.-S.N.); Pablo_Argueso@MEEI.HARVARD.EDU (P.A.); 2 Department of Ophthalmology, Harvard Medical School, Boston, MA 02114, USA; 3 Generation Bio, Cambridge, MA 02142, USA; 4 Department of Pathology, Harvard Medical School, Boston, MA 02115, USA

**Keywords:** EMCN, glycosylation, angiogenesis, VEGF, VEGFR2, mucin

## Abstract

Endomucin (EMCN) is the type I transmembrane glycoprotein, mucin-like component of the endothelial cell glycocalyx. We have previously shown that EMCN is necessary for vascular endothelial growth factor (VEGF)-induced VEGF receptor 2 (VEGFR2) internalization and downstream signaling. To explore the structural components of EMCN that are necessary for its function and the molecular mechanism of EMCN in VEGF-induced endothelial functions, we generated a series of mouse EMCN truncation mutants and examined their ability to rescue VEGF-induced endothelial functions in human primary endothelial cells (EC) in which endogenous EMCN had been knocked down using siRNA. Expression of the mouse full-length EMCN (FL EMCN) and the extracellular domain truncation mutants ∆21-81 EMCN and ∆21-121 EMCN, but not the shortest mutant ∆21-161 EMCN, successfully rescued the VEGF-induced EC migration, tube formation, and proliferation. ∆21-161 EMCN failed to interact with VEGFR2 and did not facilitate VEGFR2 internalization. Deletion of COSMC (C1GalT1C1) revealed that the abundant mucin-type *O*-glycans were not required for its VEGFR2-related functions. Mutation of the two *N*-glycosylation sites on ∆21-121 EMCN abolished its interaction with VEGFR2 and its function in VEGFR2 internalization. These results reveal ∆21-121 EMCN as the minimal extracellular domain sufficient for VEGFR2-mediated endothelial function and demonstrate an important role for *N*-glycosylation in VEGFR2 interaction, internalization, and angiogenic activity.

## 1. Introduction

Angiogenesis, the process by which new blood vessels arise from existing venules through budding and sprouting, plays an essential role in growth and development, wound healing, and repairing as well as in numerous physiological conditions [1]. In addition, angiogenesis is a hallmark of many pathogenic processes including tumor growth and metastasis, chronic inflammation, and a number of ocular pathologies [2,3]. While several factors are involved in the regulation of new blood vessel growth, vascular endothelial growth factor A (VEGF) has been shown to play a central role. Among the three tyrosine kinase receptors for VEGF, VEGF receptor 1 (VEGFR1), 2 (VEGFR2), and 3 (VEGFR3), VEGFR2 is the receptor that mediates a majority of VEGF’s biological actions.

VEGF binding to VEGFR2 initiates the formation of a multiprotein complex that includes VEGFR2 co-receptors [4], like neuropilin (NRP) and heparan sulfate proteoglycans (HSPGs), as well as integrins and endoglin [5]. Of all these associated proteins, the role of neuropilin in VEGFR2 signaling has been the best characterized. Upon VEGF binding, VEGFR2 homodimerizes and is phosphorylated on multiple tyrosine residues (Y951, Y1175, Y1059, and Y1214). Consequently, a number of signaling cascades are activated to mediate proliferation, migration, tube formation, and permeability, among other activities [6]. Activated VEGFR2 complexes with NRP1 and then is internalized through endocytosis [7]. The presence of the PDZ-binding domain of Nrp1 guides the internalized VEGFR2 to the Rab5/Rab11 pathway [8,9]. There is also evidence that VEGFR2 can be internalized through macropinocytosis [10]. Internalized VEGFR2 is either recycled back to the plasma membrane through fast or slow recycling pathways or is shuttled to lysosomes for degradation [11]. VEGF signaling is tightly regulated at all of these levels, including by receptor expression, by interactions with co-receptors or auxiliary proteins, and by the rate of internalization. For example, SCUBE2 (signal peptide-CUB-EGF domain-containing protein 2), CD146, basigin (CD147), and the urokinase plasminogen activator receptor (uPAR) [12,13,14,15] directly interact with VEGFR2 and regulate its activation, signaling, and functions. Small GTPase ARF6 controls VEGFR2 signal output via modulating its trafficking. Guanine nucleotide exchange factors (GEF) ARNO and GEP100 activate ARF6 independently. VEGFR2 interaction with ARNO activates ARF6 and stimulates VEGFR2 internalization, whereas interaction between VEGFR2 and GEP100 promotes VEGFR2 recycling via coreceptor binding. Intervening in either pathway inhibits VEGFR2 signal output [16].

The endothelial glycocalyx, which is comprised of proteoglycans and glycoproteins, extends from the luminal surface of vascular endothelium. The glycocalyx functions in cell–cell recognition, communication, and adhesion, and has been shown to play a role in various aspects of vascular homeostasis [17] and endothelial functions [18,19,20,21]. Glycosylation is one of the most common posttranslational modifications of proteins in general and of glycocalyx proteins in particular, and glycosylated proteins have been shown to play an important role in angiogenesis [14,22]. For instance, disruption of α2,6-sialylation leads to the loss of PECAM-VEGFR2 complexes, increases VEGFR2 internalization and VEGFR-dependent signaling, and impairs tumor angiogenesis through enhanced endothelial apoptosis [23]. Mucins, heavily glycosylated proteins with extended conformations, are important components of the glycocalyx [24] and have been shown to be involved in signal transduction by interacting with growth factor receptors or extracellular domain-mediated ligand binding [25,26]. Moreover, recent research indicates that mucins can strongly influence the shape and protrusions of the plasma membrane, thus having broad consequences on membrane processes, ranging from absorption and secretion to cellular communication, signaling, and motility [27].

Endomucin (EMCN) is a type I integral membrane glycoprotein expressed apically by capillary and venous endothelial cells (ECs). EMCN consists of 261 amino acids with an extracellular domain (ECD) that is highly *O*-glycosylated at serine and threonine residues and has several potential *N*-glycosylation sites. The *O*-glycans that are attached to these residues lead to a highly extended and rigid ECD. We have shown that EMCN interacts with VEGFR2 and regulates VEGFR2 internalization and its subsequent signaling [21]. In this study, we examined the structural elements of EMCN that are necessary for VEGF-induced VEGFR2 signaling. We have defined the minimum length of the EMCN ECD that interacts with VEGFR2 and is sufficient for its internalization. Moreover, we characterized the contribution of *O*-glycans and *N*-glycans on EMCN’s ECD in VEGF-induced endothelium functions.

## 2. Materials and Methods

### 2.1. Cell Culture

Primary human retinal endothelial cells (HRECs) P3 were purchased from Cell Systems (ACBRI 181). HRECs were used within passages five through ten. HRECs were cultured on dishes pre-coated with 0.2% gelatin in phosphate-buffered saline (PBS) for 30 min at 37 °C in EGM-2 BulletKit medium (Lonza, Basel, Switzerland, #CC-3162) supplemented with 2% fetal bovine serum (FBS) (Atlanta Biologicals, Flowery Branch, GA, USA), 2 mM l-glutamine (Lonza, #17-605E), and 100 U/mL penicillin–100 μg/mL streptomycin (Lonza, #17-602E). Cells were maintained at 37 °C with 5% CO_2_.

### 2.2. Reagents

Non-targeting control siRNA (siCtrl, #D-001810-01-05) and siRNA directed against EMCN (siEMCN, #L-015860-01-0005) were purchased as SMART pools (Dharmacon, Lafayette, CO, USA). Dharmafect 1 transfection reagent (Dharmacon, #T-2001-02) was used for cell culture studies. VEGF165 (#293-VE-010) was purchased from R&D Systems (Minneapolis, MN, USA). Primaquine bisphosphate (PQB, #160393-1G), paraformaldehyde (4% PFA, VWR #AAJ61899-AP), Tween-20 (#X251-07), and Phosphatase inhibitor cocktail (#4906845001) were purchased from Sigma (St Louis, MO, USA). Sulfo-NHS-SS-Biotin (1859385), DTSSP (21578), avidin agarose (#S1258122), monomeric avidin agarose (#20228), and D-Biotin (#29129) were purchased from Life Technologies (Carlsbad, CA, USA). Laemmli’s SDS Sample Buffer (#BP-110R) was purchased from Boston Bio Products (Ashland, MA, USA). Lysis Buffer (#9803S) and protease inhibitors (#5871S) were purchased from Cell Signaling (Beverly, MA, USA). Plasmid and adenovirus of mouse-truncated mutants were ordered from VectorBuilder (Chicago, IL, USA). Basically, sequence of mEMCN FL, Δ21-81, Δ21-121, and Δ21-161 were cloned into PAdEasy vector with CMV promoter through SgrDl. A Myc tag and a mCherry tag were added to the *C*-terminal of the construct, and 3xGGGGS linker was inserted between the Myc tag and mCherry tag.

### 2.3. siRNA Knockdown

HRECs were seeded at 70% confluence one day prior to siRNA transfection. siEMCN (50 nM, #L-015860-01-000) or siCtrl (50 nM, #D-001810-01-05) were incubated with Dharmafect 1 transfection reagent in OPTIMEM (Life Technologies, #51985034) at room temperature for 30 min to allow complex formation. siRNA was added in EGM-2 complete media supplemented with 2% FBS in the absence of penicillin-streptomycin. The following day, culture media were changed and cells were incubated for an additional 24 h before experiments.

### 2.4. Adenoviral Overexpression

HRECs were seeded at 70% confluence one day prior to adenoviral infection. Cells were infected with adenovirus expressing mEMCN mutants (AdmEMCN) at an multiplicity of infection (MOI) of 5 and cultured for 48 h in EGM-2 media supplemented with 2% FBS (Atlanta Biologicals, Flower Branch, GA, USA, #S11195). mCherry autofluorescence were examined using EVOS Cell Imaging System to evaluate the expression.

### 2.5. Biotin Cell Surface Isolation

Confluent HRECs were serum starved for 2 h in EBM-2 basal media. Cells were then stimulated with VEGF (10 ng/mL) in serum-free EBM-2 media supplemented with PQB (0.6 µM). Cell surface proteins were biotinylated with NHS-SS-biotin (#1859385) for 2 h at 4 °C and, then, quenched by washes in 50 mM Tris (pH 8.0) and PBS (pH 8.0), according to the manufacturer’s protocol. Cells were scraped into Tris-buffered saline (TBS) and collected by centrifugation at 1500× *g* for 5 min. Pellets were lysed in Cell Signaling Lysis Buffer and incubated with 100 µL of Avidin Agarose (#S1258122) rotating for 1 h at room temperature. Samples were washed four times in Wash Buffer (20 mM Tris-HCl, pH 6.8, 0.5% Tween-20) with protease and phosphatase inhibitors and were centrifuged at 1000× *g* for 1 min. Cell surface proteins were eluted with Laemmli’s SDS Sample Buffer (#BP-110R) with 100 mM dithiothreitol (DTT), boiled at 95 °C for 10 min, and processed for western blot analysis. Membranes were incubated with antibodies against VEGFR2 (1:1,000, Cell Signaling #55B11), hEMCN (1:300, #ab45771), or mouse anti-CD31 (1:1,000, Cell Signaling #89C2) as a loading control for cell surface fractions. Glyceraldehyde 3-phosphate dehydrogenase (GAPDH) or a-tubulin was used as loading control for whole cell lysate. For VEGFR2 internalization, the ratio of uninternalized VEGFR2 was calculated as VEGFR2 remaining on cell surface following VEGF stimulation divided by cell surface VEGFR2 in the unstimulated control. The ratio of internalized VEGFR2 equaled to 1 minus the ratio of uninternalized VEGFR2.

### 2.6. Migration Assay

HRECs were transduced with adenovirus expressing EMCN mutants at MOI 5 after reaching confluence in 12-well plate. siRNA targeting the endogenous EMCN with non-targeting siRNA as control was applied. HRECs were scratched using a P200 pipette tip 48 h after transfection. Cell debris was removed by washing with PBS. Serum-free EBM-2 basal medium (Lonza, #CC-3162) with or without 10 ng/mL VEGF was added into each well. Images of the wound edges were taken using EVOS at time 0 h and 15 h after the scratch. Migration was quantified as the closure percentage in the wound area/time and averaged for two locations per well from a triplicate set of samples for each experimental condition. The images were analyzed using Image J (National Institutes of Health and the Laboratory for Optical and Computational Instrumentation).

### 2.7. Cell Proliferation Assay

HRECs were transduced with adenovirus overexpressing EMCN-truncated mutant and siRNA targeting EMCN or non-targeting siRNA as control. HRECs were seeded in 24-well plates at 2 × 104/well and serum-starved EBM-2 basal media (Lonza, #CC-3162) 48 h after adenovirus transfection or were grown in serum-free EBM-2 basal medium (Lonza, #CC-3162) with or without VEGF (10 ng/mL) and counted using a Coulter Counter every 24 h.

### 2.8. Tube Formation Assay

Endogenous EMCN was knocked down using siRNA, and the overexpression of truncated mutants was achieved by adenoviral expression. Basement membrane extract (BME) (50 μL;, Cultrex, Gaithersburg, MD, USA) was added into 96-well plates 30 min prior to the experiment and incubated in 37 °C to solidify. Serum-starved HRECs were plated (2 × 10^4^) and treated with or without 10 ng/mL VEGF. The total length of tubes and segments formed were quantified using Image J Angiogenesis Analyzer plug-in 3 to 6 h after VEGF stimulation.

### 2.9. Immunoprecipitation

HRECs were plated in 150-mm dishes at confluence in EGM-2 complete media supplemented with 2% FBS for 48–72 h. Cells were scraped into PBS and collected by centrifugation at 1500× *g* for 5 min. Pellets were washed with cold PBS and lysed in hypotonic lysis buffer (10 mM Tris pH 7.5, 1 mM Ethylenediaminetetraacetic acid (EDTA), 1 mM MgCl_2_, and 1 mM CaCl_2_) with protease inhibitors, vortexed briefly to obtain a homogeneous cell suspension, and then centrifuged for 10 min at 16,000× *g* at 4 °C. The supernatant containing cytosolic proteins was carefully removed, and solubilization buffer (50 mM Tris-HCL, 100 mM NaCl, 5 mM EDTA, 0.5% Triton-X 100, 0.5% NP-40, pH 7.5) with protease inhibitors was added. Cell surface lysates were incubated with mouse anti-Myc (1:25, Cell Signaling #9B11) and mouse IgG (Santa Cruz, Dallas, TX, USA, sc-2025) overnight rotating at 4 °C. The following day, samples were centrifuged and beads were washed in lysis buffer followed by washes in PBS with 0.1% Tween. Bound proteins were eluted by incubation with Laemmli’s SDS Sample Buffer (# BP-110R) with 100 mM DTT, boiled at 95 °C for 10 min, and processed for western blot analysis. Membranes were incubated with antibodies against rabbit anti-human VEGFR2 (1:1,000; Cell Signaling #55B11) and rabbit anti-Myc (1:1,000; #2278S). VEGFR2 intensity was normalized to each of the corresponding mutants on beads, detected by Myc intensity, and then compared to that of the FL mEMCN.

### 2.10. Western Blot

Cells were lysed with buffer containing protease inhibitors (Roche, Basel, Switzerland) and a phosphatase inhibitor cocktail (1:100, Sigma). Protein concentration was determined by the BCA (Bicinchoninic Acid) Protein Assay (Thermo Scientific, Waltham, MA, USA, #23227). Proteins separated on SDS-PAGE gels were transferred to nitrocellulose membranes (VWR, #27376-991) and then probed with appropriate antibodies. Membranes were incubated with appropriate secondary antibodies and developed by fluorescence LI-COR Odyssey (LI-COR). Equal loading and transfer were determined by re-probing the membranes for housekeeping proteins. Densitometry analyses were performed by Image J or using Image Studio 2.0 (LI-COR). Arbitrary units of fluorescence were converted to fold-change compared to the control.

### 2.11. Immunocytochemistry-Based Internalization

HRECs were serum starved for 2 h in EBM-2 media and incubated with goat anti-VEGFR2 (R&D Systems, #AF357) or rat anti-EMCN (1:200, ab45771) at 4 °C for 1 h, followed by the addition of BSA or VEGF (10 ng/mL) for 30 min at 37 °C. Cells were fixed in 4% PFA for 5 min at room temperature and permeabilized using 0.1% Triton. Fluorescent secondary antibodies Alexa 594-labeled donkey anti-goat (1:300, Life Technologies, #A-11058) and Alexa 488-labeled donkey anti-mouse (1:300, Life Technology, #A-21202) were used for immunocytochemistry (ICC). All experiments were conducted in the presence of PQB (0.6 µM) to prevent receptor recycling. Images of cells were taken using Zeiss Axioscope (Oberkochen, Germany). Intracellular fluorescence intensity was quantified using Adobe Photoshop CC (Adobe Inc., San Jose, CA, USA) and normalized to the total number of cells per viewing field, as an indication of receptor internalization. Five images per cover slip (two coverslips) were imaged, analyzed, and averaged. Quantification reflects three independent experiments.

### 2.12. Genomic Deletion of COSMC

Ribonucleoprotein delivery of CRISPR/Cas 9 was applied. The Gene Knockout Kit v2 from Synthego with three spatially coordinated synthetic sgRNAs targeting exon 2 of the *COSMC* gene was used to improve the knockout efficiency. Non-targeting was used as control. The sequences of sgRNAs were guide #1 CUUCUUUGUUAGGAGCUUGU; guide #2 GGACACAUUAGGAUUGGUCA; and guide #3 GAAAGCAGCUCCUUUUUGAA. In brief, 3 μM sgRNA and 20 μM Cas 9 protein were introduced in HRECs through reverse transfection according to the Lipofectamine CRISPRMAX (Thermo Fisher, Waltham, MA, USA, CMAX00001) protocol.

### 2.13. Enzymatic Removal of N-glycans

HRECs were infected with adenovirus expressing mEMCN mutants (AdFL, AdΔ21-81, AdΔ21-121, and AdΔ21-161) at an MOI of 5 and cultured for 48 h in EGM-2 media supplemented with 2% FBS (Atlanta Biologicals, #S11195). Cells were scraped and pellet by centrifugation at 1000× *g* for 5 min using a table centrifuge. PNGase F (New England Biolabs, Ipswich, MA, USA, P0704S) digestion was performed as according to the manufacturer’s instructions and analyzed by western blot. In brief, cells pellets were prepared from HRECs expressing mEMCN mutants and 1 μL of PNGase F was added into 20 μL denaturing reaction and incubated in 37 °C for 1 h before western blot.

### 2.14. Statistical Analysis

All values are expressed as mean ± SEM. Statistical analysis was performed using a 2-tail unpaired t-test or one-way ANOVA post hoc test (Prism 7, GraphPad Software Inc., San Diego, CA, USA). A *p* value < 0.05 was considered statistically significant. Each experimental condition was conducted in at least triplicate, and all experiments were independently repeated at least three times.

## 3. Results

### 3.1. Δ21-121 EMCN Rescues VEGF-Induced Functions in HREC Lacking Endogenous EMCN

**Figure 1:**
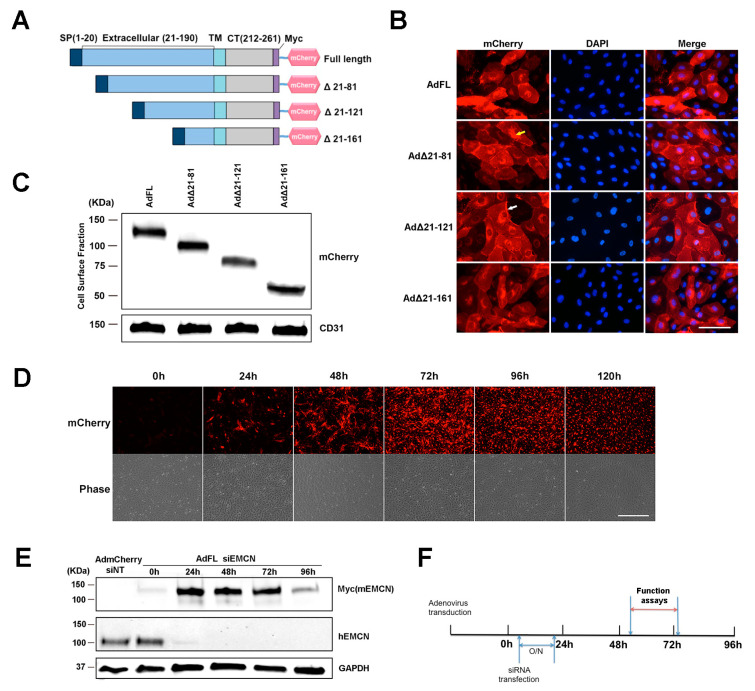
Endomucin (EMCN) mutants: constructs, expression, and experimental design. (**A**) Schematic representation of the full-length (FL) murine EMCN protein and the truncation mutants. SP, signal peptide; TM, transmembrane domain; CT, cytoplasmic tail; mCherry, fluorescence tag. (**B**) Human retinal endothelial cells (HRECs) were transduced overnight with adenoviruses expressing EMCN-truncated mutants. Autofluorescence of mCherry was examined 48 h post-infection. Scale bar: 100 um. Expression of all of the constructs was detected in the cytoplasm (indicated by yellow arrow) as well as on the cell surface (indicated by white arrow). (**C**) Adenoviruses expressing EMCN-truncated mutants were transduced into HRECs, and cell surface proteins were labeled by biotinylation and analyzed by western blot using antibodies against mCherry and CD31. The FL as well as all of the mutants were detected at the cell surface at the expected molecular weights. (**D**) HRECs were transduced with adenoviruses expressing EMCN truncated overnight, and mCherry fluorescence and phase contrast was examined every 24 h. Scale bar: 200 μm Expression was detected as early as 24 h, peaked at 72 h, and declined thereafter. (**E**) Cells lysates of HRECs in which endogenous hEMCN had been knocked down using siRNA and overexpressing full-length mouse EMCN was collected every 24 h. and protein levels of Myc-tagged (Myc mEMCN) and hEMCN were examined by western blot. GAPDH was used as loading control. Endogenous hEMCN was effectively knocked down at 24–96 h whereas exogenously expressed mEMCN was highly expressed at 24–72 h. (**F**) Experimental design for subsequent studies shows that HRECs were transduced with adenoviruses overexpressing mEMCN mutants, and one day later, endogenous hEMCN was knocked down using siRNA. In vitro functional assays, vascular endothelial growth factor receptor 2 (VEGFR2) internalization and co-IP were conducted 48–72 h post-transfection.

The ECD of EMCN is comprised of 190 amino acids. Constructs of mouse EMCN (mEMCN) with a full-length ECD (FL EMCN) and mutants with truncated ECD including full-length EMCN (FL EMCN), Δ21-81 EMCN, Δ21-121 EMCN, and Δ21-161 EMCN were generated (Figure 1A) and include a signal peptide (SP, amino acids 1–20), a Myc tag, and a mCherry tag at the *C*-terminus. The efficiency of transduction was over 90% as examined by the autofluorescent signal of mCherry. DAPI (4′,6-diamidino-2-phenylindole) was included to label the nuclei. Protein expression of the different mEMCN constructs was detected by mCherry autofluorescence in both the cytoplasm and on the cell surface (Figure 1B). Because all of the constructs included a signal sequence, we suspect the mEMCN mutants in the cytoplasm are largely localized to membranes, but resolution at the light microscopic level does not allow us to draw that conclusion. Biotinylation was used to further demonstrate cell surface protein expression of all the truncation mutants as well as to characterize the different mEMCN mutants (Figure 1C). The varying levels of cell surface expression for each mEMCN mutants is shown in Figure S1. FL EMCN, Δ21-81 EMCN, Δ21-121 EMCN, and Δ21-161 EMCN were detected at the relative molecular weights of approximately 130 KDa, 100 KDa, 75 KDa, and 50 KDa, respectively.

In order to determine the minimum size of the EMCN ECD sufficient for VEGF-induced endothelial functions, endogenous human EMCN (hEMCN) in primary HRECs was knocked down using siRNA and individual mEMCN mutants were expressed via adenovirus transduction. The optimal time course for these studies was first determined by expressing FL EMCN. Cells were incubated for 24 h with the adenovirus, and the peak FL EMCN expression was detected 48 to 96 h post-transduction by immunostaining (Figure 1D). Neither the siRNA directed against the hEMCN nor the antibodies cross-react with mEMCN (Figure 1E). The expression of total endogenous hEMCN and FL EMCN (detected by its Myc tag) at different time points is shown in Figure 1E. Expression of endogenous hEMCN protein was suppressed by over 95% from 24 to 96 h. Thus, studies were conducted under the knockdown of endogenous hEMCN and expression of the various mEMCN ECD truncation mutants as shown in Figure 1F.

**Figure 2:**
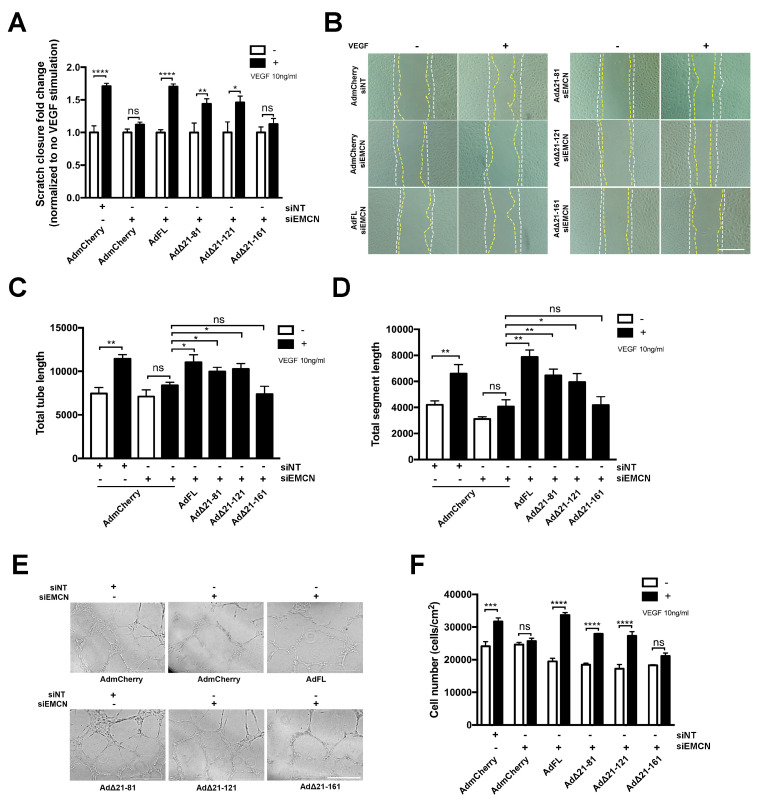
Δ21-121 EMCN is the minimal truncation mutant to rescue vascular endothelial growth factor (VEGF)-induced migration, tube formation, and cell proliferation of primary human endothelial cells. (**A**) HRECs were transduced with adenoviruses expressing the FL and different EMCN mutants individually, and endogenous hEMCN was knocked down using siRNA with non-targeting siRNA (siNT) as control. Then, VEGF-stimulated closure percentage was normalized to that without VEGF for each treatment group. EMCN knockdown abolished VEGF-induced migration, and Δ21-161EMCN failed to rescue, unlike other mutants. (**B**) Representative images of cell migration: The white line indicates the initial wound area (0 h), and the yellow line marks the wound area after 15 h. Plus and minus indicates the addition of VEGF. Scale bar: 100 um (**C**,**D**) Tube formation by HREC at 6 h was recorded, and the total tube length and total segment length were quantified using Image J angiogenesis plug-in in a masked fashion. The FL EMCN, Δ21-81, and Δ21-121 all rescued VEGF-induced tube formation whereas Δ21-161 did not. (**E**) Representative images for the tube formation assay. Scale bar: 400 µm. (**F**) HRECs were incubated in serum-free media with and without 10 ng/mL VEGF and cell number following 48 h of incubation quantified. The FL EMCN, Δ21-81, and Δ21-121 all rescued VEGF-induced HREC proliferation whereas Δ 21-161 did not. All data = mean ± SEM, ns, not significant, * *p* < 0.05, ** *p* < 0.01, *** *p* < 0.001 and **** *p* < 0.0001 by 2-tail unpaired t-test, *n* ≥ 3.

We next determined if the different mEMCN mutants could rescue VEGF-induced migration, tube formation, and/or cell proliferation in HRECs lacking endogenous EMCN. As expected, compared to the siNT control, EMCN knockdown inhibited VEGF-stimulated cell migration and expression of the control mCherry construct did not rescue cell migration (1.71 ± 0.05 versus 1.12 ± 0.04, *p* < 0.005). On the other hand, expression of FL EMCN, Δ21-81 EMCN, and Δ21-121 EMCN rescued cell migration following VEGF stimulation, compared to mCherry control (1.7 ± 0.04, 1.43 ± 0.08, 1.46 ± 0.96, versus 1.12 ± 0.04, respectively, *p* < 0.05 for all, *n* = 8). In contrast, expression of Δ21-161 EMCN was unable to rescue VEGF-induced HREC migration (1.13 ± 0.087 versus 1.12 ± 0.04) (Figure 2A,B).

The ability of the various mutants to rescue in vitro VEGF-induced tube formation assay was examined [27]. EMCN silencing inhibited both total tube formation and total segment formation (Figure 2C–E). Expression of FL EMCN, Δ21-81 EMCN, and Δ21-121 EMCN but not Δ21-161 EMCN restored VEGF-stimulated tube formation in HRECs compared to mCherry control (Figure 2C–E). Cell proliferation, another crucial component of angiogenesis, was examined by counting cell number 48 h post-VEGF stimulation. Consistent with previously observed cell functions, FL EMCN, Δ21-81 EMCN, and Δ21-121 EMCN expression also rescued cell proliferation whereas Δ21-161EMCN expression did not (Figure 2F).

### 3.2. Δ21-161 EMCN Does Not Interact With VEGFR2 or Rescue VEGFR2 Internalization

**Figure 3:**
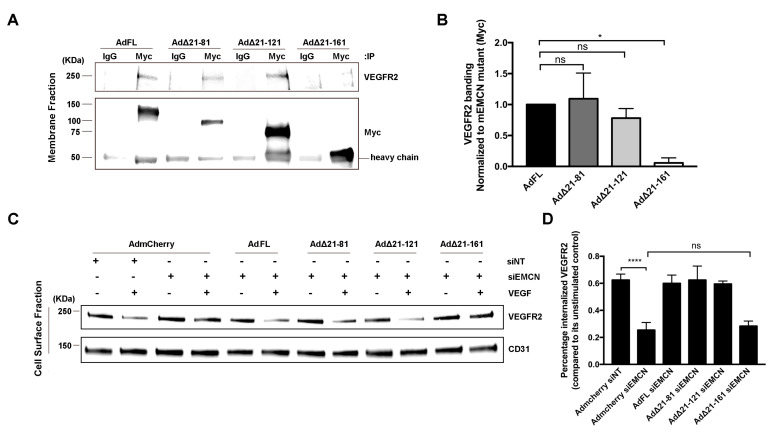
Δ21-161 EMCN does not interact with VEGFR2 or rescue VEGFR2 internalization. (**A**,**B**) Membrane proteins of HRECs lacking endogenous hEMCN and overexpressing the different Myc-tagged mEMCN constructs were extracted and subjected to co-immunoprecipitation (co-IP) using anti-Myc antibody, and the protein levels of VEGFR2 and the different mEMCN mutant proteins were examined by western blot. The FL EMCN, Δ21-81, and Δ21-121 all co-IP’ed with VEGFR2 whereas Δ 21-161 did not. (**C**,**D**) To assess VEGFR2 internalization, cell surface proteins were labeled using NHS-SS-biotin and isolated, and protein levels of VEGFR2 and CD31 were analyzed by western blot. The FL EMCN, Δ21-81, and Δ21-121 all rescued VEGFR2 internalization; Δ21-161 did not. All data = mean ± SEM, ns, not significant, * *p* < 0.05, **** *p* < 0.0001 by 2-tail unpaired t-test, *n* = 4.

VEGF mediates a majority of its effects through VEGFR2, and we have demonstrated that EMCN interacts with VEGFR2 and modulates its internalization [20]. The interaction of the different mEMCN mutants with VEGFR2 was examined by co-immunoprecipitation (co-IP) (Figure 3A), with FL EMCN and mCherry as positive and negative controls, respectively (Figure S2). The interaction of ∆21-81 EMCN and ∆21-121 EMCN with VEGFR2 was comparable to that for FL EMCN (1.1 ± 0.29, 0.8 ± 0.11 vs 1.00 ± 0.05, respectively); however, VEGFR2 interaction with ∆21-161EMCN was significantly reduced compared to FL EMCN (0.01 ± 0.0819 vs 1.00 ± 0.02, *p* < 0.01) (Figure 3A,B).

Next, the effect of the various EMCN ECD mutants on VEGFR2 was examined. Cell surface expression of VEGFR2 following VEGF treatment was determined with NHS-SS biotin labeling and extraction using avidin beads (Figure 3C,D). EMCN knockdown suppressed VEGFR2 internalization compared to control (0.25 ± 0.03 vs 0.62 ± 0.025, *p* < 0.001) (Figure 3C,D). Expression of FL EMCN, ∆21-81 EMCN, or ∆21-121 EMCN rescued VEGFR2 internalization in HRECs deficient in endogenous EMCN compared to mCherry control (0.6 ± 0.04, 0.62 ± 0.06, 0.6 ± 0.01 vs 0.25 ± 0.03, respectively, *p* < 0.001 for all). Consistent with its inability to rescue VEGF-induced cell migration and tube formation, ∆21-161 EMCN failed to rescue VEGFR2 internalization upon VEGF stimulation, compared to mCherry control (0.28 ± 0.02 versus 0.25 ± 0.03).

### 3.3. O-Glycosylation of the EMCN ECD Is Not Necessary for VEGFR2 Internalization

**Figure 4:**
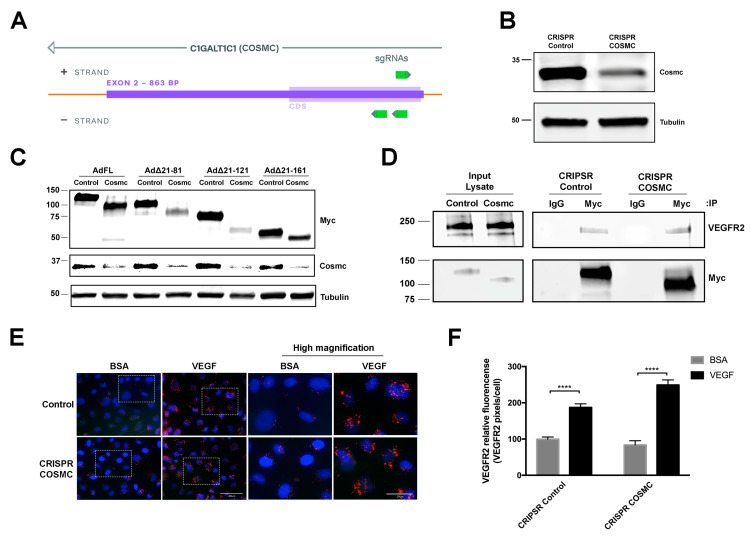
*O*-glycosylation of EMCN is not required for modulating VEGFR2 signaling. (**A**) Design of the CRISPR-Cas9 for targeted COSMC knockout in HREC. (**B**) Whole cell lysates from CRIPSR control and COSMC knockout (CRIPSR Cosmc) were extracted and processed for western blot, which showed approximately 70% reduction in COSMC protein levels; *n* = 3 (**C**) Whole cell lysate samples were collected 72 h post-transduction. Molecular weight of the Myc-tagged FL EMCN and EMCN mutants, with and without mucin-type *O*-glycosylation, were examined by western blot using anti-Myc tag antibody. Protein levels of COSMC revealed a significant reduction of COSMC at the protein level, and α-tubulin was used as loading control, confirming the reduction of COSMC. Examination by western blot of FL EMCN, ∆21-81 EMCN, ∆21-121 EMCN, and ∆21-161 EMCN revealed molecular weight reductions of ~30 kDa, ~25 kDa, ~20 kDa, and ~10 kDa, respectively, consistent with reduced *O*-glycosylation; *n* = 3 (**D**) The levels of VEGFR2 co-IP’ed with EMCN in CRIPSR control HRECs or COSMC knockout HRECs (CRIPSR Cosmc) and examined by western blot indicate that the lack of *O*-glycosylation did not impact the interaction of EMCN and VEGFR2; *n* = 3 (**E**,**F**) To examine VEGFR2 internalization, CRIPSR control HRECs or COSMC knockout HRECs were stimulated with VEGF or BSA and VEGF2 tracked with an antibody that recognizes the VEGFR2 extracellular domain (ECD). VEGF stimulated VEGFR2 internalization in both cells, indicating that *O*-glycosylation of EMCN is not necessary for VEGFR2 internalization. Boxes areas in the left-hand panel are shown magnified in the right-hand panels. Lower magnification, scale bar: 40 μm, higher magnification, scale bar: 20 μm. (**F**) The levels of Internalized VEGFR2 were quantified as VEGFR2 pixels divided by the number of cells per images. Data = mean ± SEM, **** *p* < 0.0001 by 2-tail unpaired t-test, *n* = 6.

NetOGlyc 4.0 Server (http://www.cbs.dtu.dk/services/NetOGlyc/) predicts 57 potential *O*-glycosylation sites on hEMCN ECD and 58 *O*-glycosylation sites on mEMCN, with 63% of the *O*-glycosylation sites overlapping. Thus, the ECD of EMCN is heavily *O*-glycosylated, whereas only five are predicted *N*-glycosylation sites. To further investigate the structural elements involved in EMCN-VEGFR2 interaction, we examined how the role *O*-glycosylation in the ECD is involved in EMCN regulation of VEGFR2 signaling. COSMC (C1GALT1C1) is the unique and sole chaperone of T-synthase, which is the only enzyme that galactosylates the Tn antigen [28]. T-synthase is the only enzyme that galactosylates the Tn antigen (GalNAcα1-Ser/Thr-R) to form core 1 Galβ1–3GalNAcα1-Ser/Thr (T antigen) during mucin-type *O*-glycan biosynthesis [29]. Total cell lysate from pooled CRIPSR (clustered regularly interspaced short palindromic repeats) COSMC HRECs revealed a 70% reduction of Cosmc at the protein level (Figure 4B,C). The FL EMCN and the different mouse truncation mutants were expressed in CRIPSR COSMC and control HRECs constructs to examine the effect of COSMC deletion on protein *O*-glycosylation (Figure 4C). We suspect that the 50k-Da band observed in AdFL and Ad∆21-121 transfected cells is a degradation product.

The role of *O*-glycosylation in the interaction with VEGFR2 was examined by co-IP in FL EMCN (Figure 4D). No significant change in VEGFR2 interaction was detected between EMCN with reduced *O*-glycosylation and control EMCN. Since FL mEMCN contains all of the *O*-glycosylation sites, it was not necessary to conduct additional studies to examine the *O*-glycosylation in individual mutants. Moreover, the ability of EMCN with reduced *O*-glycans to facilitate VEGFR2 internalization was measured utilizing ICC-based VEGFR2 internalization assay. As we have previously shown, loss of EMCN prevented VEGFR2 internalization [21] (Figure S3). Control HREC with non-targeting sgRNA and wildtype EMCN exhibited significant VEGFR2 internalization following VEGF stimulation compared to BSA-treated controls (99.4 ± 6.3 vs 187.2 ± 9.9, *p* < 0.001), and similarly, CRIPSR COSMC HRECs also showed significant VEGFR2 internalization following VEGF stimulation (83.7 ± 11.6 vs 250.2 ± 13.8, *p* < 0.001) (Figure 4E,F), indicating that *O*-glycosylation of the FL EMCN ECD was not required for VEGFR2 internalization.

### 3.4. N-Glycans of the EMCN ECD Are Essential for Its Role in VEGFR2 Function

**Figure 5:**
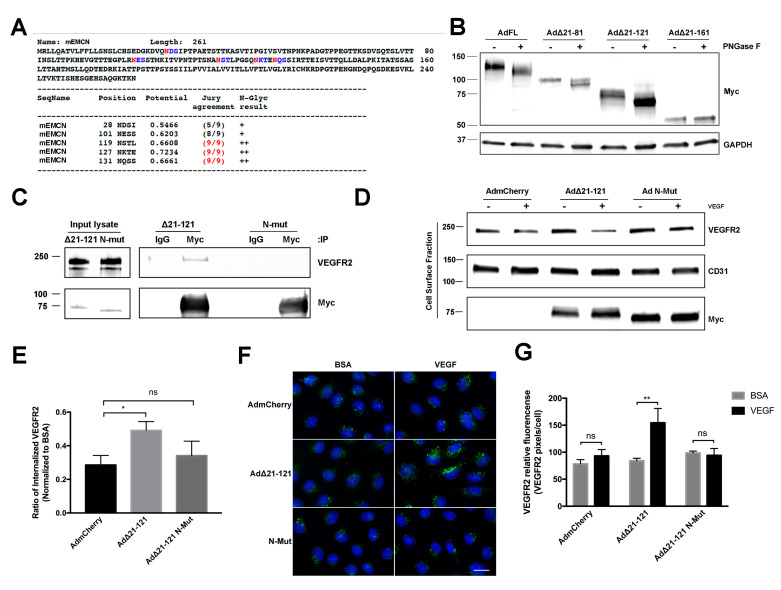
*N*-glycans on EMCN ECD are essential for EMCN’s role in VEGFR2 signaling. (**A**) Potential *N*-glycosylation sites (asparagine, N, red) on full-length mEMCN predicted by NetNGlyc 1.0 Server. (**B**) Molecular weight of FL EMCN, Δ21-81 EMCN, Δ21-121 EMCN, and Δ21-161 EMCN with and without PNGase F digestion were examined by western blot; GAPDH was included as loading control. Observed molecular weights are in aligned with predicted sizes. (**C**) Membrane proteins of HRECs (lacking endogenous hEMCN) expressing Myc-tagged EMCN Δ21-121 EMCN or Δ21-121 EMCN with *N*-glycan sites mutated (N-Mut) were co-IP’ed for using anti-Myc and examined by western blot. The mutant lacking *N*-glycosylation did not bind to VEGFR2. (**D**) HRECs lacking endogenous hEMCN and expressing either Δ21-121 EMCN or N-Mut were used to examine cell surface expression of VEGFR2 by biotinylation. As seen for the co-IP, the Δ21-121 lacking *N*-glycosylation was unable to rescue VEGFR2 internalization compared to the normally glycosylated EMCN mutant. (**E**) Quantification shows reduced VEGFR2 internalization in the absence of *N*-glycosylation. * *p* < 0.05 by 2-tail unpaired t-test, *n* = 4. (**F**) Immunocytochemical assay to detect internalized VEGFR2 proteins in HRECs stimulated with VEGF or BSA: Consistent with the findings in Figure 5D,E, Δ21-121 EMCN was able to rescue VEGFR internalization where the *N*-glycosylation mutant was not. Scale bar: 20 μm. (**G**) Internalized VEGFR2 was quantified as total VEGFR2-positive pixels divided by the total number of cells per images. Data = mean ± SEM, ns, not significant, ** *p* < 0.01 by 2-tail unpaired t-test, *n* = 6.

The NetNGlyc 1.0 Server (http://www.cbs.dtu.dk/services/NetNGlyc/) predicted five *N*-glycosylation sites (28N, 101N, 119N, 127N, and 131N) on the mEMCN ECD (Figure 5A). Whole cell lysates of HRECs expressing FL EMCN, ∆21-81 EMCN, ∆21-121 EMCN, and ∆21-161 EMCN treated with PNGase F to enzymatically release the *N*-glycans yielded reduction in molecular weights of the Myc-tagged EMCN mutants following the PNGase F digestion were, as expected, approximately 20 KDa, 15 KD, 8 KDa, and 0 KDa, respectively (Figure 5B). A similar 25-kDa reduction in hEMCN molecular weight was observed after *N*-glycan removal (Figure S4). Consistent with the lack of any predicted *N*-glycosylation sites, no change in molecular weight was detected for the ∆21-161 EMCN mutant.

There are two predicted *N*-glycosylation sites on ∆21-121 EMCN (Figure 5A), the shortest truncation mutant, which we have shown can interact with and modulate the function of VEGFR2 (Figure 5A). To investigate the role of *N*-glycans in EMCN function, the two predicted *N*-glycosylation sites of ∆21-121 EMCN were mutated from asparagine to alanine (N127A and N131A) to generate the *N*-glycosylation deficient mutant (N-Mut). The interaction with VEGFR2 was completely abolished in the N-Mut compared to the wildtype ∆21-121 EMCN by the cell surface biotinylation/IP/western assay (Figure 5C), demonstrating that *N*-glycosylation of the ECD of ∆21-121 EMCN is required for its interaction with VEGFR2. The effect of ∆21-121 EMCN or N-Mut on VEGFR2 internalization in HRECs lacking endogenous EMCN was examined and quantified by western blot (Figure 5D,E). Expression of ∆21-121 EMCN rescued VEGFR2 internalization compared to mCherry, while expression of N-Mut failed to rescue VEGFR2 internalization compared to mCherry. ICC-based VEGFR2 internalization (Figure 5F,G) was consistent with the results above and revealed that the N-Mut failed to rescue the VEGFR2 internalization after VEGF stimulation in HRECs lacking the endogenous EMCN (98.67 ± 3.4 vs 94.25 ± 12.6, *p* > 0.5) (Figure 5 F,G).

## 4. Discussion

We have previously demonstrated that the endothelial-selective glycoprotein EMCN is a key regulator of VEGF signaling. EMCN knockdown using siRNA prevents VEGF-stimulated cell proliferation, migration, and tube formation in HRECs in vitro and significantly impairs retinal vessel development in vivo [30]. In addition, we have shown that EMCN plays a role in these activities by facilitating the internalization of ligand-bound VEGFR2 [21]. Here, we undertook studies to examine the structural features of the EMCN molecule that are required for VEGF-induced ECs functions to further reveal the molecular mechanism by which EMCN regulates VEGF signaling.

As an important component of the glycocalyx, mucin backbones have heavily glycosylated ectodomains, forming the characteristic bottlebrush molecular structures [26]. Like all mucins, the ECD of mEMCN (amino acids 19–190), which comprises more than 70% of the molecule (amino acids 19–190), is highly glycosylated. Indeed, EMCN protein core is predicted to account for approximately 27.5 KDa of the molecular weight while the remainder (as measured by western-blot) is due to *O*- and *N*-glycosylation. To determine the role of EMCN ECD, mEMCN truncation mutants were designed according to the predicted glycosylation pattern. Though glycosylation is known to affect the membrane localization and stability of glycoproteins [31], all mutants were glycosylated and detected on the cell surface. FL EMCN, the positive control, rescued VEGF-stimulated EC functions in cells deficient in endogenous hEMCN. mEMCN truncation mutants, Δ21-81 EMCN, and Δ21-121 EMCN rescued the VEGF-stimulated angiogenesis functions compared to vehicle construct mCherry (negative control). On the other hand, the shortest mutant Δ21-161 EMCN failed to facilitate VEGF-mediated ECs functions, indicating that the amino acids 121-190 of the EMCN ECD are sufficient for most, if not all, VEGF-stimulated downstream functions. While the difference between Δ21-121 EMCN and Δ21-161 EMCN in their ability to modulate VEGFR2 signaling could suggest an important functional role for amino acids 121-161 of the ECD, our data exclude the domains shared between the two mutants. The Δ21-161 EMCN mutant includes 28 amino acids of the ECD, the transmembrane and the intracellular domains, which we were unable to express separately. However, these data using Δ21-161 EMCN indicate that the intracellular domain alone would not be sufficient to rescue VEGF-induced functions.

Upon binding VEGF, VEGFR2 is internalized via the clathrin-mediated pathway [32] or macropinocytosis [9] and is then either recycled back to the plasma membrane or targeted to the proteasome for lysosomal degradation. Clathrin-independent endocytosis, like endophilin-A2 dependent internalization, has been reported [33]. VEGFR2 signaling has been shown to continue following endocytosis, which is reported to be necessary for successful VEGF signal transduction and activation of ERK1/2 in endothelium [34]. Thus, modulating VEGR2 endocytosis can greatly impact VEGF-induced functions. For example, silencing clathrin-associated sorting protein, Dab2, impairs VEGFR2 internalization and reduces downstream signal transduction [35]. Regulator of calcineurin 1.4 (RCAN 1.4) regulates agonist-stimulated VEGFR-2 internalization, modulating VEGF-stimulated endothelial cell migration by establishing endothelial cell polarity [36]. VEGFR2 associates with other receptors and/or glycoproteins such as neuropilin-1 (NRP1), heparin sulfate proteoglycans, and endoglin [7,37]. NRP1, a VEGFR2 co-receptor, interacts with VEGFR2 and is internalized with VEGFR2 to guide its recycling [4]. EMCN interacts with VEGFR2 and is essential for its internalization, but unlike NRP1, EMCN itself is not internalized along with VEGFR2 following VEGF stimulation [21]. Here, we show that Δ21-121 EMCN interacts with VEGFR2 and is sufficient to support both the interaction of EMCN with VEGFR2 as well as VEGFR2 internalization. X-ray crystallographic analysis of VEGFR2 suggests that its ECD extends around 200 Å from the cell surface [38,39]. While the structure of EMCN has not been characterized, it is reasonable to predict that the high level of glycosylation causes EMCN to project rigidly from the cell surface, similar to other mucins. Assuming 3.5 Å per amino acid, the full-length ECD of EMCN is estimated to be roughly 600 Å [40,41], whereas Δ121-121 EMCN, the smallest mutant that can rescue VEGF-induced functions, would be estimated to be approximately 300 Å. In contrast, the Δ21-161 mutant that was unable to rescue, would be about 175 Å, possibly too short to interact optimally with VEGFR2.

Mucin-type *O*-linked glycosylation is so important in many biological processes [42] since C1GalT1 (T synthase) is the only enzyme that glycosylates the Tn antigen (GalNAcα1-Ser/Thr-R) to form core 1 Galβ1-3GalNAcα1-Ser/Thr (T antigen) [43]. The absence of the required chaperone, Cosmc, results in immature and truncated *O*-glycans that lack biological function [28,44]. In order to study the role of EMCN *O*-glycans in VEGFR2-EMCN interaction and VEGFR2 internalization, we generated Cosmc knockout HRECs using CRISPR/Cas 9 and showed that mucin *O*-glycosylation contributes approximately 30 KDa to the molecular weight of the mouse EMCN. As there are more than 50 predicted *O*-glycosylation sites on the EMCN ECD, this indicates that, while mucin-type *O*-glycans are abundant, at an average molecular weight of approximately 600 Da, their chains are relatively short. Importantly, in spite of their abundance, co-IP data showed that *O*-glycan chains on EMCN are not necessary for EMCN-VEGFR2 interaction. Consistent with these findings, an immunocytochemistry-based assay showed that impaired *O*-glycosylation on EMCN did not prevent VEGF-induced VEGFR2 internalization.

We next turned our attention to the possible contribution of *N*-glycans. *N*-glycan precursors are synthesized in the endoplasmic reticulum as a branched structure on a lipid anchor. *N*-glycosidase F (PNGase F) cleaves the linkage between the asparagine residue and innermost *N*-acetylglucosamine (GlcNAc) of nearly all *N*-glycans [45]. An approximate 20 KDa reduction in molecular weight was observed in hEMCN following PNGase F-induced cleavage of *N*-glycans compared to control, suggesting that, with an approximate molecular weight of 4000 Da, the *N*-glycans on EMCN are relatively long and/or highly branched. *N*-glycans are known to facilitate proper folding of nascent proteins; to enhance the protein solubility or polarity; and, most relevant to our findings, to mediate protein association in receptor/ligand complexes or sugar-specific binding proteins in the plasma membrane that mediate receptor endocytosis and trafficking [46,47,48,49,50]. For example, galectin-3 binds to *N*-linked glycans on CD146/MCAM (Melanoma Cell Adhesion Molecule) and induces CD146 dimerization and subsequent activation of AKT (Protein kinase B) signaling [51]. Galectin-1 (Gal1) delivers angiogenic signals through a glycosylation-dependent pathway involving context-dependent remodeling of complex *N*-glycans. Targeting the Gal1-*N*-Glycan could overcome resistance to anti-VEGF [52]. Our data indicate that *N*-glycans on EMCN are crucial in the EMCN–VEGFR2 interaction and internalization.

VEGF induces both angiogenesis and vascular permeability. Angiogenesis is rate limiting for tumor growth and metastasis and is a devastating component of a number of ocular pathologies such as proliferative diabetic retinopathy and wet age-related macular degeneration. While VEGFR2 is widely expressed, not only by the endothelium but also by a variety of epithelial cells and neurons [53], EMCN is specifically expressed by endothelial cells, particularly in proliferating capillaries [29]. Accordingly, EMCN represents an endothelial-specific target for modulating VEGF responses, so a deeper mechanistic understanding of its role in VEGF signaling presented here is essential for future translational development of EMCN as a potential therapeutic target.

## Supplementary Materials

The following are available online at https://www.mdpi.com/2073-4409/9/6/1413/s1, Figure S1: Cell surface expression of mEMCN mutants: Indicated EMCN mutants were expressed in HREC, and cell surface expression was examined using biotinylation followed by western blotting, Figure S2: FL EMCN interacts with VEGFR2, Figure S3: EMCN knockdown prevents VEGFR2 internalization, Figure S4: *N*-glycosylation in human EMCN.
