# Supplementary material for: Elements of the Endomucin Extracellular Domain Essential for VEGF-Induced VEGFR2 Activity
Source: Cells. 2020 Jun 5;9(6):1413. doi: 10.3390/cells9061413 (PMC7349057; doi:10.3390/cells9061413)
Supplement: Supplementary file 1 [file cells-09-01413-s001.pdf]

Supplementary Material

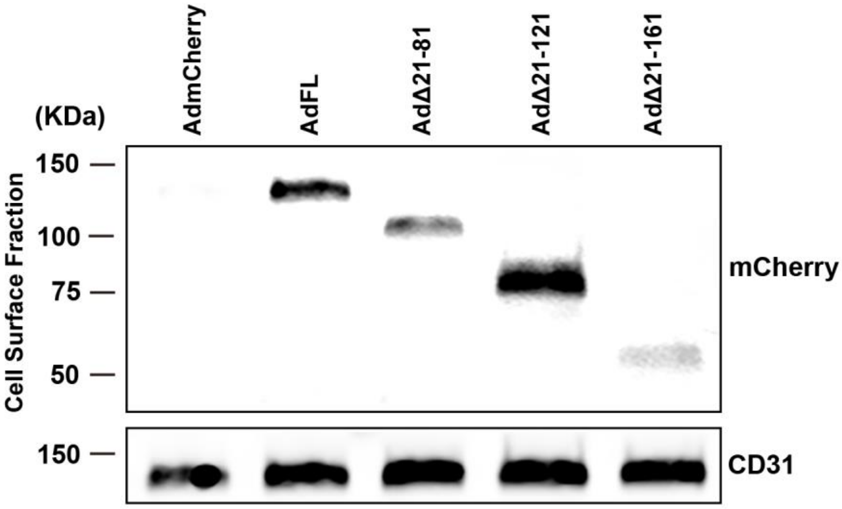

Figure S1. Cell surface expression of mEMCN mutants. Indicated EMCN mutants were expressed in HREC and cell surface expressed was examined using biotinylation followed by western blotting. The FL and the three truncation mutants were all expressed on the cell surface. Expression levels varied among different experiments as shown by comparison of this blot to that shown in Figure 1C.

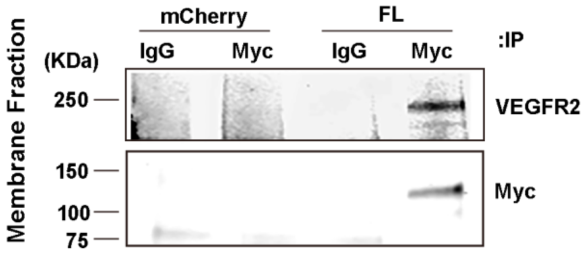

Figure S2. FL EMCN interacts with VEGFR2. Membrane proteins of HRECs lacking endogenous hEMCN and expressing FL EMCN or mCherry control were extracted and processed for co-IP. mEMCN FL interacted with VEGFR2, which served as positive control, whereas, mCherry didn't interact with VEGFR2, served as negative control.

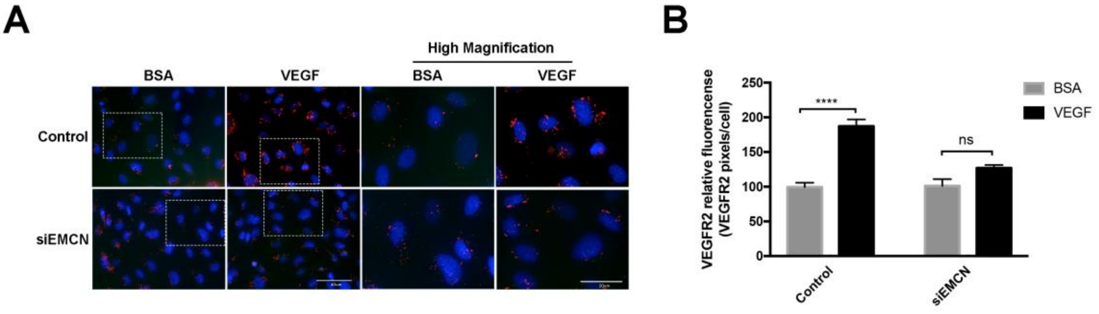

Figure S3. EMCN knockdown prevents VEGFR2 internalization. (**A**) Representative images of the VEGFR2 internalization assay in which HRECs were transfected with siRNA targeting EMCN (siEMCN) or control non-targeting siRNA and were stimulated with 10 ng/ml VEGF or BSA 30 min. EMCN knockdown abolished VEGF-induced VEGFR2 internalization. Red fluorescence indicated

internalized VEGFR2, which a reduction of internalized VEGFR2 was observed in EMCN knockdown cells following VEGF stimulation. (**B**) Internalized VEGFR2 was quantified as VEGFR2-positive pixels divided by the number of cells per images (DAPI). EMCN knockdown prevented VEGF stimulated VEGFR2 internalization. Data = mean ±SEM, **** $P < 0.0001$ by unpaired Student's t-test, $n = 6$ per treatment condition.

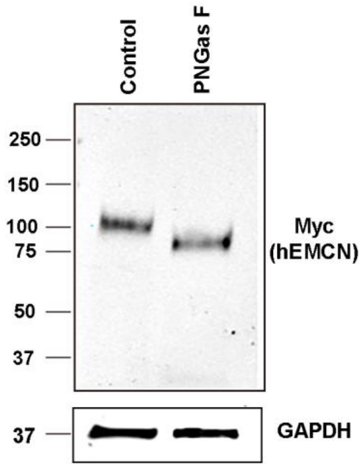

Figure S4. *N*-glycosylation in human EMCN. Changes in molecular weight of human EMCN protein extracted from HREC, with and without PNGase F digestion to remove *N*-glycans, were examined by western blot. GAPDH was included as a loading control. In line with mEMCN, the molecular weight that *N*-glycosylation contributed in hEMCN is about25 kDa.
